# Intubation Practices and Outcomes in Diverse Emergency Departments Within a Single Health System

**DOI:** 10.5811/westjem.61961

**Published:** 2026-07-28

**Authors:** Sarah Gonzalez-Bankich, Emily C. Sterrett, Tim Crittenden, Brian Burrows, Joseph Borawski, Neel Kapadia, Beiyu Liu, Emily Greenwald

**Affiliations:** * Duke University School of Medicine, Durham, North Carolina; † Division of Pediatric Emergency Medicine, Department of Pediatrics, Duke University School of Medicine, Durham, North Carolina; ‡ Duke Health Technology Solutions, Durham, North Carolina; § Duke Regional Hospital, Department of Emergency Medicine, Durham, North Carolina; || Duke University School of Medicine, Department of Emergency Medicine, Durham, North Carolina; # Duke University School of Medicine, Department of Biostatistics & Bioinformatics, Durham, North Carolina

## Abstract

**Introduction:**

Endotracheal intubation (ETI) is a life-saving procedure commonly performed in emergency departments (ED). Prior research has demonstrated significant variability in ETI experience, practices, and outcomes across EDs. Health systems often include a mix of ED environments, from large academic hospitals to community-based and freestanding EDs. However, little is known about how ETI experiences and outcomes vary within a single health system. Our objective in this study was to compare first-attempt success across three EDs representing different practice environments but within one health system.

**Methods:**

This retrospective observational study used institutional data from 1,659 ETI events between November 2021–January 2025 to compare ETI-related outcomes across three EDs representing a spectrum of settings: an academic Level I trauma center (A); a community teaching hospital (B); and a nonteaching community site (C). Our primary outcome measure was first-attempt success. We also assessed adverse airway outcomes and ETI-related patient and procedural characteristics. Chi-square tests evaluated the association of ETI-related outcomes and hospital sites.

**Results:**

Annual ED volumes were approximately 82,000 (A), 64,000 (B), and 49,000 (C), with respective ETI encounters per 1,000 patients of 5, 2, and 0.4. The community teaching hospital had the highest first-attempt success rate of 90.2% (B: 331/367; 95% CI, 87.2–93.2), followed by the academic center with 84.6% (A: 1038/1227, 95% CI, 82.6–86.6), and then by the nonteaching community site with 78.5% (C: 51/65; 95% CI, 68.5–88.5) (*P* = .01). Adverse airway outcomes occurred in 10.5% of ETIs and did not vary significantly between sites (A: 139/1,227 [11.3%]; B: 29/367 [7.9%]; C: 6/65 [9.2%], *P =* .16). The most common indication was airway protection, followed by altered mental status and cardiopulmonary arrest for all three sites. Trainees performed the majority of ETIs at hospital A (1,096/1,227; 89.3%), while attending physicians predominated at B (295/367; 80.4%) and C (64/65; 98.5%).

**Conclusion:**

We found significant variation in first-attempt success between the three ED sites, although overall success and adverse airway outcomes were comparable. These findings suggest that first-attempt success may be impacted by factors such as patient differences, role and training level of laryngoscopists, and inherent site-level differences such as patient volume and acuity.

## INTRODUCTION

### Background

Endotracheal intubation (ETI) is a life-saving procedure performed on critically ill patients in emergency departments (ED). The primary goal of the procedure is to secure the airway and provide respiratory support.^1^ First-attempt success is widely accepted as the most important outcome measure for ETI because failure to intubate on the first attempt is associated with adverse airway outcomes. Adverse airway outcomes are comprised of tracheal intubation-associated adverse events and hypoxia, which are associated with poor short- and long-term outcomes.^2^–^5^ Further, as the number of attempts increases, the incidence of adverse outcomes increases substantially.^6^

### Importance

While previous studies have compared first-attempt success rates between different departments of individual hospitals,^7^,^8^ little is known about how ETI practices and outcomes differ between academic and community EDs, especially within single health systems. Academic EDs are fully university affiliated, serve as primary training sites with a tiered team of trainees, and support clinical, educational, and research missions, whereas community EDs lack full academic affiliation, have minimal or no trainee involvement, and focus primarily on direct clinical care. Most studies looking at ETI first-attempt success include only Level I trauma or academic medical centers, which limits generalizability to community-based settings. Prior studies suggest that distinguishing between community and academic EDs is important because they differ in their operations, resources, patient populations, and outcomes.^9^

To the best of our knowledge, there is only one prior study that has characterized ETI in community EDs.^10^ While the authors found that ETI practices in community EDs tend to mirror those of academic centers, the distinction between these contexts may be important in accounting for factors such as the frequency of ETI procedures,^11^ available equipment, and presence of different levels of laryngoscopists who perform ETI.^12^–^14^ Community EDs may exist as part of a larger health system that includes academic centers or may operate independently; to date, no studies have examined ETI practices within a single health system that encompasses both academic and community EDs.

### Goals of this Investigation

The goal of this investigation was to compare ETI outcomes across three EDs that are part of a single health system but represent a spectrum of settings from an academic quaternary care and Level I trauma center to community-based hospitals. This project is part of a larger health system initiative to examine our local emergency airway performance to inform improvement efforts and drive airway excellence. We sought to characterize and understand the variation in practice across the three distinct EDs by evaluating the primary outcome of first-attempt success. We also investigated adverse airway outcomes and differences in local environment, patients, and procedural practices.

**Population Health Research Capsule**

What do we already know about this issue?

*First-attempt success in emergency department (ED) intubation varies by factors such as equipment and operator, and failed attempts increase adverse airway outcomes*.

What was the research question?

*How do endotracheal intubation (ETI) success and adverse outcomes vary across three EDs within one health system?*

What was the major finding of the study?

*First-attempt success differed by site (90.2% vs 84.6% vs 78.5%; P = .01); adverse outcomes were similar (10.5%; P = .16); confidence intervals overlap*.

How does this improve population health?

*Understanding site-level variation in airway success may inform targeted training and system strategies to improve emergency care outcomes*.

## METHODS

### Design and Setting

We conducted a retrospective observational study using data from an institutional database of emergency ETI procedures. The study was set within a three-hospital health system, each facility operating its own ED (Figure).

Hospital A is a quaternary academic medical center and designated Level I trauma center with an embedded pediatric ED (PED). It houses 1,062 inpatient beds, 172 of which are pediatric beds, and manages approximately 82,000 ED visits annually. Hospitals B and C are community hospitals. Hospital B has 388 adult inpatient beds, an 18-bed neonatal intensive care unit, and an annual ED volume of approximately 64,000 visits, while Hospital C is a smaller facility with 204 adult beds and 49,000 annual ED visits.

Resident physicians provide care at both Hospitals A and B. During the study period, there was no overlap in attending emergency physicians across the three sites, although a small number of resident physicians who trained at Hospital A subsequently joined the attending staff at Hospitals A and B. The PED at Hospital A is always staffed by fellowship-trained pediatric emergency physicians, and coverage is supplemented further by a dedicated group of general emergency physicians who devote at least 30% of their clinical practice to pediatric patients. While pediatric patients are also treated at Hospitals B and C, these sites do not have pediatric emergency physician specialists.

### Data Collection

Data was collected from an institutional dataset that captures emergency ETI events across the institution and includes discrete and retrievable data elements aligned with National Emergency Airway Registry standards. The development of this dataset was part of an institutional effort to improve airway performance. An airway taskforce composed of a multidisciplinary team of physicians and informatics specialists developed a standardized documentation tool in the electronic health record. From 2021, all airway events in the health system’s three EDs were documented using this standardized note. We considered all 1,703 documented ED ETI procedures from the three EDs from November 2021–January 2025 for inclusion in this study. We excluded 44 documented procedures (2.58% of total) because the number of attempts was not recorded. Several criteria of medical record review studies were followed, as outlined in Worster et al (2005),^15^ including training data abstractors, using clear case-selection criteria, defining variables, identifying our database, describing our sampling method, and describing management of missing data.

### Outcomes of Interest and Definitions

The primary outcome of interest was first attempt success, defined as a patient encounter with only one ETI attempt resulting in successful placement of an endotracheal tube. We also analyzed the outcome of adverse airway outcomes, which were defined as the presence of a tracheal intubation-associated adverse event and/or peri-intubation hypoxia within 20 minutes of the procedure’s start. Peri-intubation hypoxia was defined as an oxygen saturation drop below 80% in a patient who achieved a saturation of at least 90% during peri-intubation pre-oxygenation. Tracheal intubation-associated adverse events were defined as complications arising during or because of ETI; these included cardiac arrest, hypotension, mainstem intubation, inadequate sedation, dental/lip trauma, emesis, dysrhythmia, hypertension, direct airway injury, esophageal intubation, laryngospasm, pneumothorax, and malignant hyperthermia.^16^

To add further context, we sought to characterize the following patient and procedural characteristics of ETI. Patient characteristics included the following:

- ETI indication: reasons why a patient required ETI, as determined by the laryngoscopist. Each encounter may have multiple linked indications given that many indications overlap in clinical practice, among them airway protection/clearance, altered mental status, apnea/bradypnea, blood in airway, cardiac with medical cause, cardiac with surgical cause, cardiopulmonary arrest, emergency drug administration, endotracheal tube exchange, hemodynamic instability, hypercarbia, neuromuscular weakness, procedure, re-intubation after unplanned extubation, respiratory distress, respiratory failure, shock/sepsis, therapeutic hyperventilation, facial trauma, neck trauma, and upper airway obstruction.
- Airway difficulty: a yes/no subjective assessment made by the laryngoscopist at the time of ETI; typically, this reflected a patient’s anatomical features or significant injury to or blood secretions in the airway.

Procedural aspects include the following:

- Laryngoscopist characteristics: role, specialty, and level of training of personnel who performed ETI during each attempt.
- ETI technique: Two techniques were used—direct laryngoscopy, in which the operator visualizes the vocal cords directly, and video laryngoscopy, in which the operator visualizes the cords indirectly via a camera-equipped screen.

### Statistical Analysis

We summarized patients’ and procedural intubation characteristics, stratified by hospital ED, using descriptive statistics. Categorical variables were reported with frequency counts and percentages of nonmissing values. The first-attempt success rate and its 95% confidence interval were reported. We used the chi-squared test to analyze the association between first-attempt success and hospital ED and adverse airway outcomes and hospital ED. *P* values were assessed at a significance level of 0.05. We performed all statistical analyses using SAS v9.4 (SAS Institute Inc, Cary, NC) and Microsoft Excel v16.102.3 (Microsoft Corporation, Redmond, WA).

## RESULTS

### Number of Endotracheal Intubations and Patient Characteristics

Of 1,659 ETI events documented across the three EDs, nearly three-quarters occurred at Hospital A (1,227/1,659; 74%). Close to one-quarter occurred at Hospital B (367/1,659; 22.1%), with only a small percentage occurring at Hospital C (65/1,659; 3.9%) (Table 1).

More ETI occurred in male patients compared to female patients (980/1,659; 59.1% versus 678/1,659; 40.9%) across the three sites. Adults made up most patients who underwent ETI (1,540/1,659; 92.8%). The median age for adult patients was 61 years and for pediatric patients four years. The number of pediatric ETIs was small (119/1,659 [7.2%]) and varied dramatically between sites; very few pediatric ETIs occurred outside Hospital A. Overall, 341 (20.6%) ETI events were noted to have a difficult airway (Table 1). The proportion of difficult airways was highest at Hospital A, which had 275 (22.4%) (Hospital C: 12 [18.5%], Hospital B 54 [14.7%]).

### Indications for Endotracheal Intubation

The three most frequent ETI indications were consistent across the health system. The most common category was altered mental status/neurologic; the most frequent subcategory was airway protection with 67.8% of system-wide ETI events (A: 852/1,227, 69.4%; B: 230/367, 62.7%; C: 43/65, 66.2%). The second most common sub-category was altered mental status both overall (658/1,659, 39.7%) and at each hospital (A 490/1,227, 39.9%; B: 139/367, 37.9%; C 29/65, 44.6%). The second most common subcategory was hemodynamic instability and third was preintubation cardiopulmonary arrest associated with 33.3% (408/1,659) of ETI events and a similar trend at each hospital (A: 282/1,227, 23.0%; B: 115/367, 31.3%; C: 11/65, 16.9%). Airway protection (61/119, 51.3%) and respiratory (47/119, 39.5%) were the most common indications for pediatric intubations.

All but one of the ETI associated with the indication of face and neck trauma took place at Hospital A. No trauma-related indications occurred at Hospital C. A full description of ETI indications is found in Table 1.

### First-Attempt Success and Overall Success

The health system ED first-attempt success rate was 85.6% (95% CI, 83.9–87.3). However, we found a statistically significant relationship between first-attempt success and each ED (*P =* .01). Hospital B achieved the highest first-attempt success rate (90.2%; 95% CI, 87.2–93.2), followed by Hospital A (84.6%; 95% CI, 82.6–86.6), and Hospital C (78.5%; 95% CI, 68.5–88.5). Overall success was over 99% for all three hospitals. Only two ETI events were ultimately unsuccessful, one resulting in death (Hospital A) and one resulting in cricothyrotomy (Hospital B).

### Adverse Airway Outcomes

There was no statistically significant difference in the rate of adverse airway outcomes among the three hospitals (*P =* .16). The health system ED rate was 10.5% (174/1,659) (Table 2). Hospital A had the highest proportion of adverse airway outcomes, with 11.3% (139/1,227) followed by Hospital C, with 9.2% (6/65) and Hospital B with the least frequent rate of 7.9% (29/367). Hypoxia was more frequent than any tracheal intubation-associated adverse event and occurred in 4.7% (78/1,659) of total ETI: 5.6% (69/1,227), at Hospital A, 1.6% (6/367) at Hospital B, and 4.6% (3/65), at Hospital C. Hypotension was the most frequent tracheal intubation-associated adverse event, occurring in 2.7% (44/1,659) of encounters. Hospital B had the highest rates of hypotension, occurring in 4.6% (17/367) of encounters, followed by Hospital A in 2.1% (26/1,227), and 1.5% (1/65) at Hospital C.

Peri-intubation cardiac arrest occurred in 1.4% (24/1,659) of encounters. Return of spontaneous circulation was achieved in 63% (15/24) of these events, including 78% (14/18) at Hospital A, 20% (1/5) at Hospital B, and 0% (0/1) at Hospital C. Death occurred in 37% (9/24) of peri-intubation cardiac arrests: 22% (4/18) at Hospital A, 80% (4/5) at Hospital B, and 100% (1/1) at Hospital C. Other differences in less frequent adverse airway outcomes are listed in descending order of frequency in Table 2.

### Laryngoscopists

Most final attempts were performed by physician trainees (residents and fellows) (1,160/1,659; 69.9%). However, the majority (89.3%) were performed at hospital A (1,096/1,227) (B: 64/367, 17.4%). Among physician trainees who performed final-attempt ETIs, most were at the PGY-3 level, followed by PGY-2 and PGY-1. By contrast, emergency attending physicians performed only 6.8% (83/1,227) final-attempt ETIs at Hospital A. They performed most procedures at Hospital B and Hospital C, accounting for 80.4% (295/367) and 98.5% (64/65), respectively.

Additionally, respiratory therapists, nurse practitioners, and physician assistants performed comparatively small numbers of final-attempt ETIs. Respiratory therapists played the largest role at Hospital B, performing 2.2% (8/659) of final-attempt ETIs. Nurse practitioners and physician assistants played the largest role at Hospital A, performing 1.8% (22/1,227) of final-attempt ETIs. All EDs had mechanisms to call for additional personnel in the case of difficult ETIs. This included mechanisms to activate an emergency response from anesthesia and surgical teams. At Hospitals B and C, the standard procedure was for one person to attempt the ETI then page anesthesia or surgical teams if they were unsuccessful or anticipated further difficulties. At Hospital A, it was common practice for several people in the ED (for example, escalating from resident to attending) to attempt the ETI prior to calling for additional help. Hospital A was the only hospital to have any final-attempt ETIs performed by anesthesiologists, critical airway team members, trauma service members, and intensivists—all of these occurred via hospital A’s critical airway response team. A full description of ETI laryngoscopists is found in Table 3.

### Visualization Technique and Devices

Direct video laryngoscopy was the predominant visualization technique across all three EDs, used in 1,405 ETIs (84.7%) compared with 148 ETIs (8.9%) via direct laryngoscopy. Video laryngoscopy rate was highest at Hospital A (1,085/1,227; 88.4%), followed by Hospital B (283/367; 77.1%) and Hospital C (37/65; 65.9%). Hospital C had the greatest proportion of direct laryngoscopy (21/65; 32.3%), compared with Hospital B (61/367; 16.6%) and Hospital A (66/1,227; 5.4%). All three EDs had equivalent access to both video laryngoscopy and direct laryngoscopy equipment.

### Pediatric Experience

During the study period, of the 119 ETI events in pediatric patients (< 18 years of age), 117 (98.3%) occurred at Hospital A, which has an embedded PED, and two occurred at Hospital C (1.7%). The overall first-attempt success rate in d pediatric patients was 72.3% (86/119). This was 72.6% (85/117) at Hospital A and 50% (1/2) at Hospital C. The rate of pediatric adverse airway outcome was 20.2% (24/119), with 11.8% of pediatric encounters experiencing hypoxia (14/119). Difficult airway at the time of ETI was reported in 21.8% of pediatric patients (26/119).

## DISCUSSION

In this retrospective, observational study of ETI events across three EDs within a single health system, we found a statistically significant relationship between first-attempt success and hospital site, but no significant relationship between adverse airway outcome and hospital. Patient and procedural characteristics also varied broadly between sites. Importantly, the rate of ETI between sites was variable, with Hospital A performing 5/1,000 patient visits, Hospital B 2 /1,000, and Hospital C just 0.4 per 1,000. This likely reflects baseline patient acuity with sicker patients being treated at the large academic tertiary-care center compared with the two community hospitals and is further evidenced by the higher admission rates seen at Hospital A (Figure 1). However, this study was not designed to determine the causes of site-level differences in procedural frequency.

Despite differences in ETI rates, overall success rate for emergency airway management was > 99%, which is consistent with previously described patterns in adult and pediatric emergency airway rescue.^17^,^18^ The overall FAS rate (85.6%) across all three sites also aligns with prior literature,^19^–^22^ but masks site-level variation. Several factors may contribute to these differences.

Hospital B had the highest first-attempt success rate and reflected a clinical environment where experienced attendings perform most procedures (80%) but are still required to supervise and teach trainees who perform 20% of ETIs. This is compared to Hospital A, where physician trainees performed nearly 90% of ETIs, and Hospital C, where attendings performed all ETIs. These patterns suggest that differences in laryngoscopist experience may be associated with site-level differences in first-attempt success, which supports the need for further study on how procedural context and laryngoscopist role relate to airway outcomes in different ED environments. Prior studies have shown that skill proficiency in ETI is associated with the average number of procedures performed annually, with attending physicians who perform < 3 ETIs per year or supervise < 5 having lower proficiency,^23^ which may be relevant when interpreting the variation observed across our three study sites. Future work should more directly address the role of skill maintenance for laryngoscopists in diverse ED settings.

The distribution of adverse airway outcomes similarly supports the importance of site-level analysis, even in the absence of statistically significant differences. The overall adverse airway outcome rate was 10.5%, and rates did not differ significantly between hospitals (*P* = .16). These findings suggest relative system-level consistency in overall adverse airway outcomes, although because we did not conduct statistical analysis on individual adverse airway outcomes, local variation in patient characteristics, and procedural context may still be clinically important and worth further exploration.

The academic ED, Hospital A, had the highest proportion of documented difficult airways. This likely reflects the higher acuity and medical complexity of patients at this site. Further study is needed to understand whether the differences in difficult airways represent statistical significance. Future work may also focus on clinically meaningful differences and how to prepare community sites within health systems for difficult airways and to see whether these differences can be mitigated by strategies such as rotation or training of attending physicians through the academic, higher acuity site.

While Hospital A had the highest proportion of adverse airway outcome, it also had the lowest rate of death from peri-intubation cardiac arrest with 73.7% (14/19) of peri-intubation cardiac arrest events resulting in death (compared with 80% [4/5] at Hospital B and 100% [1/1]) at Hospital C. Given the small number of cardiac arrest events at the latter two hospitals, these differences should be interpreted cautiously. It is possible that the finding reflects differences in patient population, resuscitation practices, or chance. For example, due to the finding that Hospital A had the most medically complex patients (as indicated by the higher admission rate and more cases of “difficult” airways), higher rates of return of spontaneous circulation could reflect multiple factors such as better site-level resuscitation competency, earlier recognition and intervention in peri-intubation cardiac arrest or teams that are more practiced in caring for critically ill patients.^24^ As our institutional database grows, this may be an area for future statistical analysis rather than firm interpretation at this stage.

Hypoxia was the most common adverse airway outcome across the health system. The higher rate of hypoxia at Hospital A may be partially explained by differences in patient population, including higher baseline acuity, as evidenced by the higher admission rate. Additionally, Hospital A had the most pediatric encounters. Hypoxia was documented in 12% (14/117) of pediatric encounters at Hospital A, compared with 5.2% (58/1110) among adult patients. This higher rate of peri-intubation hypoxia is consistent with prior pediatric airway literature and may reflect increased susceptibility to hypoxia in this population.^16^

While adverse airway outcomes such as cardiac arrest often reflect underlying patient instability, other complications such as inadequate sedation, dental/lip trauma, mainstem intubation, and airway injury may be more preventable. Nearly all occurred at Hospital A. These events are rare; therefore, it is possible that the higher rate at Hospital A reflects the higher number of ETI procedures. Nonetheless, these findings may help identify areas for additional review and quality improvement.

Overall, the similarity in adverse airway outcome rates across the three hospitals suggests some amount of system-level consistency in airway management. Nonetheless, targeted site-specific interventions remain valuable based on clinical context. For example, academic centers such as Hospital A may benefit from interventions that improve readiness specifically for high-risk ETIs. Examples include pre-intubation assessment and checklist tools and “difficult airway” alerts in patient charts, which can improve airway management outcomes. 25–27 In contrast, community EDs such as Hospital C that are staffed only by attending physicians and see a much lower volume of ETI procedures may benefit from interventions such as high-fidelity simulation for high stakes, low frequency airway scenarios and peri-intubation cardiac arrest and other in-service training methods that emphasize maintenance of competency.^28^ They may also benefit from protocols that rapidly mobilize additional team members to augment the leaner team structure that many community centers use compared with academic centers.^29^

Regarding visualization technique, our findings demonstrate a predominance of video laryngoscopy across all three EDs, with particularly high use at Hospital A. These findings align with broad consensus in emergency medicine favoring video laryngoscopy to achieve higher first-attempt success rates, particularly among less experienced laryngoscopists.^1^,^30^–^39^ The variation between hospitals may reflect differences in operator level of training. Hospital C, where 100% of laryngoscopists are attending physicians, had the highest use of direct laryngoscopy. By comparison, Hospital A, where laryngoscopists are primarily residents, had the lowest use of direct laryngoscopy. Because each hospital stocks identical airway equipment, these differences are unlikely to be explained by resource availability; however, in this study we were unable determine reasons for equipment choices made by laryngoscopists. Given the large evidence-base supporting video laryngoscopy for improved patient outcomes as cited above, future work should explore what factors influence the choice of laryngoscopy technique in different ED contexts.

Our data also offer insight into the pediatric ETI experience across a health system. The overall pediatric first-attempt success rate was 72.3%, lower than the overall cohort, but consistent with what has been previously found.^40^–^42^ Further, the pediatric adverse airway outcome rate was 20.2%, nearly double the overall adverse event rate of 10.5%. Pediatric encounters account for a large proportion of the adverse airway outcomes at Hospital A (Of 139 adverse airway outcomes, 17.3% were in pediatric patients despite pediatric patients only accounting for 9.5% of total ETIs.) This is consistent with prior literature, which demonstrates that first-attempt success tends to be lower and adverse airway outcome s tend to be higher in pediatric patients due to their unique anatomy and physiology,^43^,^44^ with the most difficult and complicated adverse airway outcomes occurring in infants and toddlers.^16^,^45^,^46^ Further, prior studies show that pediatric patients who present to PEDs tend to be sicker than those who present to general EDs.^47^ Taken together, these findings support continued attention to pediatric airway prepareness within health systems.

## LIMITATIONS

Our study has several limitations. Firstly, our data depend on direct input by individuals, and we were unable to verify the accuracy of documentation via video event review or other methods. Further, while it is institutional protocol for all ETI events to be documented using the same standardized procedure note that feeds into the database used in this study, it is possible that some events were documented using a different method or were not documented at all; however, since billing for the ETI procedure is linked to the institutional documentation, we believe the rate of mis-documentation is likely very low if not zero. Additionally, the procedure note contains definitions for each piece of data collected to ensure consistency across documenters. For example, our note defines an “attempt” as passing the laryngoscope beyond the teeth, but the definition varies across the literature on first-attempt success, making it difficult to directly compare our results with those studies that use a different definition.

Being this study is retrospective electronic health record-driven analysis, we also acknowledge potential under-reporting of time-delayed tracheal intubation-associated adverse events. For example, we found that rates of hypotension were < 4% at each site, which is much lower compared with prospectively collected data on tracheal intubation-associated adverse events. Because certain tracheal intubation-associated adverse events, such as hypotension, may not occur or be recognized until several minutes after the procedure is complete, this creates a systematic bias toward under-reporting of these time-delayed events, compared with events that occur more contemporaneously with the procedure such as right mainstem intubation, hypoxia, and direct airway injury.

Lastly, as noted above, we considered the embedded PED and general ED at Hospital A as a single site, which may limit the generalizability of the findings given the variation in pediatric and adult patients.

## CONCLUSION

The goal of this study was to investigate site-level variation in emergency endotracheal intubation across three different EDs that are part of a single health system. We found evidence of significant variation in first-attempt success between the sites, although rates of adverse events were comparable and overall success rates were over 99% across the hospitals. These findings suggest that procedural success is impacted by factors such as patient differences, role and training level of laryngoscopists, and inherent site-level differences such as patient volume and acuity. Further analysis and intervention should be tailored to the characteristics of specific sites.

## Figures and Tables

**Figure:**
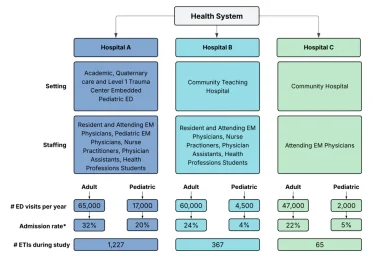
Descriptions of health system hospitals with three distinct emergency departments in a study examining variations in practice by evaluating first-attempt success for endotracheal intubations, *Hospital B and Hospital C do not have pediatric units. The admission rate for these hospitals represents transfer to a pediatric hospital, mostly Hospital A for admission. *ED*, emergency department; *EM*, emergency medicine; *ETI*, endotracheal intubation.

**Table 1:** Demographic characteristics of patients undergoing endotracheal intubation, by each health system emergency department site and overall.

Total ETIs, N (%)	Overall 1,659 (100%)	Hospital A 1,227 (74%)	Hospital B 367 (22.1%)	Hospital C 65 (3.9%)
Patient characteristics:				
Sex, N (%)				
Male	980 (59.1%)	732 (59.7%)	213 (58.0%)	35 (53.8%)
Unknown	1 (0.1%)	1 (0.1%)	0 (0.0%)	0 (0.0%)
Age, N (%)				
Pediatric	119 (7.2%)	117 (9.5%)	0 (0.0%)	2 (3.1%)
Adult, age known	1538 (92.7%)	1108 (90.5%)	367 (100.0%)	63 (96.9%)
Adult, age unknown	2 (0.12%)	2 (0.16%)	0 (0.0%)	0 (0.0%)
Median adult	61	60	63	65
Median pediatric	4	4	-	3.5
Difficult airway				
Anticipated today	341 (20.6%)	275 (22.4%)	54 (14.7%)	12 (18.5%)
History of	26 (1.6%)	22 (1.8%)	4 (1.1%)	0 (0.0%)
ETI Indication				
AMS/Neurologic:				
Airway protection^**^	1125 (67.8%)	852 (69.4%)	230 (62.7%)	43 (66.2%)
Altered mental status	658 (39.7%)	490 (39.9%)	139 (37.9%)	29 (44.6%)
Neuromuscular weakness	6 (0.4%)	4 (0.3%)	2 (0.5%)	0 (0.0%)
Hemodynamic instability				
Cardio/pulmonary arrest	408 (24.6%)	282 (23.0%)	115 (31.3%)	11 (16.9%)
Hypoxia	276 (16.6%)	174 (14.2%)	85 (23.2%)	17 (26.2%)
Hemodynamic instability	112 (6.8%)	76 (6.2%)	29 (7.9%)	7 (10.8%)
Shock/sepsis	56 (3.4%)	32 (2.6%)	21 (5.7%)	3 (4.6%)
Cardiac - medical	49 (3.0%)	33 (2.7%)	15 (4.1%)	1 (1.5%)
Respiratory				
Respiratory failure	257 (15.5%)	169 (13.8%)	70 (19.1%)	11 (16.9%)
Respiratory distress	129 (7.8%)	81 (6.6%)	37 (10.1%)	11 (16.9%)
Hypercarbia	100 (6.0%)	68 (5.5%)	28 (7.6%)	4 (6.2%)
Apnea/bradypnea	68 (4.1%)	43 (3.5%)	22 (6.0%)	3 (4.6%)
Upper airway obstruction	8 (0.5%)	5 (0.4%)	1 (0.3%)	2 (3.1%)
Trauma*				
Blood in airway	101 (6.1%)	86 (7.0%)	8 (2.2%)	7 (10.8%)
Trauma - face	60 (3.6%)	59 (4.8%)	1 (0.3%)	0 (0.0%)
Unknown	44 (2.7%)	38 (3.1%)	6 (1.6%)	0 (0.0%)
Procedure	30 (1.8%)	27 (2.2%)	3 (0.8%)	0 (0.0%)
Trauma - neck	17 (1.0%)	17 (1.4%)	0 (0.0%)	0 (0.0%)
Therapeutic hyperventilation	4 (0.2%)	3 (0.2%)	1 (0.3%)	0 (0.0%)
Other				
ETT exchange	16 (1.0%)	9 (0.7%)	7 (1.9%)	0 (0.0%)
Other	11 (0.7%)	8 (0.7%)	3 (0.8%)	0 (0.0%)
Emergency drug administration	5 (0.3%)	5 (0.4%)	0 (0.0%)	0 (0.0%)
Reintubation after unplanned extubation	1 (0.1%)	1 (0.1%)	0 (0.0%)	0 (0.0%)
Cardiac - surgical	1 (0.1%)	1 (0.1%)	0 (0.0%)	0 (0.0%)

Values are presented as counts with corresponding percentages, calculated using each hospital’s total number of ETIs. Age is categorized into pediatric (< 18 years of age) and adult (≥ 18) groups. Median age is provided for each cohort. For ETI indication, multiple indications could be linked to each ETI event as determined by the laryngoscopist. This differs from prospective airway registries where there is a single indication assigned to each ETI event. “Difficult airway today” refers to intubations assessed as difficult during the documented ETI event. “Difficult airway history” reflects documented history of airway difficulty in the patient chart.

* Airway protection indication includes both trauma patients as well as medical reasons, although the majority (almost 100%) of ETI for trauma occurred at Hospital A.

*AMS*, altered mental status; *ETI*, endotracheal intubation; *ETT*, endotracheal tube.

**Table 2:** Adverse airway outcomes by hospital and overall.

Adverse Airway Outcome	Overall	Hospital A	Hospital B	Hospital C	*P* value
Any AAO	174 (10.5%)	139 (11.3%)	29 (7.9%)	6 (9.2%)	.16
Hypoxia	78 (4.7%)	69 (5.6%)	6 (1.6%)	3 (4.6%)	
Tracheal intubation-associate adverse event					
Hypotension	44 (2.7%)	26 (2.1%)	17 (4.6%)	1 (1.5%)	
Cardiac arrest	25 (1.5%)	19 (1.5%)	5 (1.4%)	1 (1.5%)	
Death	10 (0.6%)	5 (0.5%)	4 (1.1%)	1 (1.5%)	
Mainstem intubation	18 (1.1%)	17 (1.4%)	1 (0.3%)	0 (0.0%)	
Inadequate sedation	14 (0.8%)	13 (1.1%)	1 (0.3%)	0 (0.0%)	
Dental/lip trauma	3 (0.2%)	2 (0.2%)	1 (0.3%)	0 (0.0%)	
Emesis no aspiration	5 (0.3%)	4 (0.3%)	1 (0.3%)	0 (0.0%)	
Dysrhythmia	5 (0.3%)	5 (0.4%)	0 (0.0%)	0 (0.0%)	
Hypertension	4 (0.2%)	4 (0.3%)	0 (0.0%)	0 (0.0%)	
Direct airway injury	3 (0.2%)	3 (0.2%)	0 (0.0%)	0 (0.0%)	
Esophageal intubation	2 (0.1%)	1 (0.08%)	0 (0.0%)	1 (1.5%)	
Emesis with aspiration	2 (0.1%)	1 (0.08%)	0 (0.0%)	1 (1.5%)	
Laryngospasm	1 (0.06%)	1 (0.08%)	0 (0.0%)	0 (0.0%)	
Pneumothorax	1 (0.06%)	0 (0.0%)	1 (0.3%)	0 (0.0%)	
Malignant hyperthermia	0 (0.0%)	0 (0.0%)	0 (0.0%)	0 (0.0%)	

Listed in order of overall health system descending frequency. Values represent the number and percentage of total endotracheal intubation (ETI) encounters. Percentages were calculated using the total number of ETIs at each hospital and overall. Multiple adverse events could be linked to a single ETI.

*AAO*, adverse airway outcome.

**Table 3:** Role of the final intubation attempt operator by hospital and overall.

Final-attempt laryngoscopist	Hospital A	Hospital B	Hospital C	Overall
Resident / Fellow	1,096 (89.3%)	64 (17.4%)	0 (0.0%)	1,160 (69.9%)
PGY 1	296 (24.1%)	1 (0.3%)	0 (0.0%)	297 (17.9%)
PGY 2	287 (23.4%)	60 (16.3%)	0 (0.0%)	347 (20.9%)
PGY 3	443 (36.1%)	2 (0.5%)	0 (0.0%)	445 (26.8%)
PGY 4	44 (3.6%)	0 (0.0%)	0 (0.0%)	44 (2.7%)
PGY 5 +	6 (0.5%)	0 (0.0%)	0 (0.0%)	6 (0.4%)
Unknown PGY	19 (1.5%)	1 (0.3%)	0 (0.0%)	20 (1.2%)
Emergency attending	83 (6.8%)	295 (80.4%)	64 (98.5%)	442 (26.6%)
Nurse practitioner/physician assistant	22 (1.8%)	0 (0.0%)	1 (1.5%)	23 (1.4%)
Respiratory therapist	11 (0.9%)	8 (2.2%)	0 (0.0%)	19 (1.1%)
Anesthesiologist	6 (0.5%)	0 (0.0%)	0 (0.0%)	6 (0.4%)
Trauma service	5 (0.4%)	0 (0.0%)	0 (0.0%)	5 (0.3%)
Critical airway team	2 (0.2%)	0 (0.0%)	0 (0.0%)	2 (0.1%)
Intensivist	1 (0.1%)	0 (0.0%)	0 (0.0%)	1 (0.1%)
Other	1 (0.1%)	0 (0.0%)	0 (0.0%)	1 (0.1%)
Unknown	3 (0.2%)	2 (0.5%)	1 (1.5%)	6 (0.4%)

Resident postgraduate year (PGY) levels are broken out separately. “Unknown (PGY)” reflects cases where resident training level was not specified. The “Resident / Fellow” row includes all PGY levels and unknowns. Percentages are calculated using total endotracheal intubation events at each hospital and overall.

*PGY*, postgraduate year.
